# Prioritization of Farm Animal Welfare Issues Using Expert Consensus

**DOI:** 10.3389/fvets.2019.00495

**Published:** 2020-01-10

**Authors:** Fiona C. Rioja-Lang, Melanie Connor, Heather J. Bacon, Alistair B. Lawrence, Cathy M. Dwyer

**Affiliations:** ^1^Jeanne Marchig International Centre for Animal Welfare Education, The Royal (Dick) School of Veterinary Studies, University of Edinburgh, Easter Bush Campus, Midlothian, United Kingdom; ^2^Animal Behaviour and Welfare, Animal and Veterinary Sciences Group, Scotland's Rural College, Edinburgh, United Kingdom

**Keywords:** animal welfare, Delphi method, cattle, sheep, pigs, poultry, behavior, health

## Abstract

Prioritization of animal welfare issues can help identify which areas most require research funding and raise awareness of best practices. A modified Delphi method was used to obtain expert opinion on the highest priority welfare issues for UK farmed livestock. Fifty-eight UK-based experts were recruited onto the study, with a minimum of 3 years experience of working with either cattle, pigs, poultry, or small ruminants (12–16 experts per group). Experts were chosen to represent a broad range of opinions. Two rounds of surveys were conducted online using Online Survey, and the final round was an in-person workshop with 21 experts. In the first survey, experts were provided with a comprehensive list of species-specific welfare issues derived from the literature. Participants were asked to score each welfare issue, for (i) severity, (ii) duration, and (iii) prevalence on a 6-point Likert scale. The results of the first survey were reviewed and the welfare issues which scored a neutral-to-high response (scores 3–6) were carried forward. In round 2, participants were asked whether they agreed or disagreed with the rankings that were made from the results of round 1. The final stage of the process was a workshop, which consisted of a combination of group exercises and discussions, to reach the final consensus. Welfare priority lists were divided into two categories: severity/duration, and prevalence, to identify the priority welfare issues affecting individual animals and the population, respectively. Across all farmed species common concerns were inadequate or inappropriate nutrition, inability of stockpeople to recognize or treat welfare issues (such as pain or behavioral problems), lameness, chronic or endemic health issues, euthanasia and mortality and morbidity of neonates. Specific concerns related to behavioral restriction and damaging or abnormal behavior in pigs, poultry and dairy animals, inadequate housing for pigs and poultry, consequences of breeding decisions in pigs and poultry, and lack of access to veterinary care in sheep and beef. This Delphi process resulted in consensus on the most significant welfare challenges faced by UK livestock species and can help to guide future research and education priority decisions.

## Introduction

Animal welfare remains an area of consistent public concern, with acceptance of animal sentience enshrined in the legislation of many countries. Historically, animal welfare concerns have been directed primarily at farmed livestock, with increasing awareness and unease at the intensification and confinement of animals in the 1960s [e.g., following the publication of “Animal Machines” by Ruth Harrison in 1964 (1)]. In the United Kingdom, there is strong animal welfare legislation with good compliance generally (2). However, there are differing views of what is important for animal welfare, from different stakeholders, which may result in conflicting welfare outcomes (3, 4), and decisions on which aspects of welfare to prioritize.

Traditionally, farm animal welfare has referred to minimal acceptable standards of farm animals. However, development of animal welfare science over the last decade have started to shift from providing acceptable welfare standards that minimize animal “harms” or suffering toward providing animals with “positive” welfare (2, 5). Acceptable standards of animal welfare can be determined by legislation, which may set the minimal standard that a society is prepared to accept in food production, and by retailer and other accreditation schemes, labels or standards that provide higher levels of animal care. The inclusion of positive animal-based welfare measures, such as the ability to move freely and a positive human-animal relationship, among the proposed guiding principles for OIE animal welfare standards (6) reflects that positive welfare is now an active topic of discussion on the world stage (7). Assessing farm animal welfare concerns and improving standards is an ongoing process and one that requires significant scientific research and financial support.

The relative priority given to specific welfare issues can be determined by public concern, political interests, such as international trade or legislation, or by weighing the scientific evidence. Animal welfare science is a large field, since it embraces everything that may affect the physical and emotional state of the animal, its ability to cope and its overall quality of life (8). Governmental and funder resources for tackling animal welfare issues are often limited (9), therefore the prioritization of farm animal welfare issues can help identify which areas most require research funding and raise awareness of best practices.

There are many possible methodologies to try to rank the welfare priorities of animals. However, eliciting expert consensus has been shown to be reliable and very helpful in animal welfare science (10–12). In this study a modified Delphi method was used. The Delphi method is based on the assumption that group consensus is more representative than individual opinion. The method relies on a group of experts participating in multiple rounds of surveys in an attempt to reach consensus on an issue. The Delphi method uses “rounds” of surveys, with each round becoming more specific/focussed. The classical Delphi method is anonymous, liberating participants to speak their mind without influence of others' views or opinions.

The objective of this study was to prioritize welfare issues of cattle, pigs, poultry, and small ruminants in the UK using expert consensus. To achieve this we brought together expertise from multiple key stakeholders in animal welfare (e.g., veterinarians, academics, practitioners, farmers, voluntary, and charitable bodies, etc.) to generate consensus on the most important animal welfare issues for farmed livestock.

## Methods

All research generated from this study was approved by the University of Edinburgh's Human Ethics Review Committee (HERC). The full description of the methods can be found in Rioja-Lang et al. (13). The results presented here are part of a larger study that identified priority welfare issues for a number of managed animal species in the UK.

### Recruitment of Experts

Experts were defined as having worked in their field for more than 3 years, were based in the UK and thus assumed to be knowledgeable about UK farming systems, and were practicing veterinarians, academics, charity sector employees, farmer representatives, and policy officials with responsibility for farmed livestock.

We recruited 58 experts to the project (pigs = 15; poultry = 12; cattle = 16; small ruminants = 15). The recruitment process began with building a list of contacts of well-known experts in their field and contacting them via email and describing the study, the aims, and the Delphi procedure. Additionally, we employed a “snowball-sampling method” whereby these initial contacts were also asked to refer us to other experts in their field who would be a good addition to the study. When an expert agreed via email to participate in the study, they were then sent a consent form to sign in accordance with the HERC guidelines. The consent form also contained a more detailed description of the study objectives, protocol, and expected timeline. It was also explained to each expert that participation was anonymous (except for the workshop) and voluntary, and they were able to leave the process at any time.

### Literature Review

A review of the scientific and industry literature was conducted to construct a comprehensive list of documented welfare issues for each species. However, at every stage experts were also given an opportunity to add any other issues that they felt might be missing. These original lists generated from the literature consisted of welfare issues covering a broad spectrum of animal-based welfare outcomes, management, and resource-based risk factors (cattle = 72; pigs = 80; poultry = 81; small ruminants = 76; see Supplementary Material for full lists). For the purposes of the online surveys, the welfare issues of beef and dairy cattle were considered together, small ruminants consisted of meat and dairy goats and sheep, and the poultry group consisted of laying and spent hens, broiler birds, turkeys and ducks. During the workshop the experts re-categorized the ruminant welfare issues to “Dairy animals (cows and goats)” and considered “Sheep and Beef cattle” together.

### Questionnaire Design

Two rounds of surveys were conducted online using the Online Surveys tool (formerly Bristol Online Survey). Each round of surveys was completed anonymously at a time suitable to each expert within a 2-week window. In both rounds of online surveys, demographic data were also collected from the participants. This included: year of birth, gender, profession, highest level of education, and number of years since graduating highest level of education.

The experts were asked via email for the criteria they considered important to rank/score welfare issues. Experts agreed that welfare issues should be ranked according to three different criteria: (i) severity (defined as the likely severity associated with the welfare issue in the experts opinion), (ii) duration (defined as the likely period of time that the animal would be affected by the welfare issue in the experts opinion), and (iii) prevalence (defined as the experts perceived proportion of the population affected by the welfare issue). However, all criteria were considered equally important in prioritizing animal welfare, and therefore were all used in determining welfare ranking. During round 1 of the online surveys, experts were asked to score each welfare issue for each of the three criteria, on a 6-point Likert scale, where 1 = never/none, and 6 = always/high. An even numbered scale was chosen as this forced experts to make a decision (at least somewhat important to unimportant).

The results of the first survey were reviewed and the welfare issues which scored a median neutral-high response (scores 3–6 on the Likert scale) on any of the criteria were carried forward into round 2 as these were those that the experts ranked as being at least somewhat important. These welfare issues were then presented to the experts according to their ranking from round 1, with the highest-ranking issues (score 6) at the top of the list, and the lowest ranked issues (score 3) at the bottom. This process was completed for all issues scoring at least 3 for each criteria. Experts were asked whether they agreed or disagreed with the rankings. In instances where experts did not agree with the relative ranking, they were asked to indicate if they would like to see that issue higher or lower on the ranked lists. Agreement between experts was determined by calculating Fleiss' kappa statistics, and assessing the percentage of experts who agreed with the ranking.

### Workshop

The final stage of the Delphi process was the workshop, held at Edinburgh University in early Sept, 2018. Twenty-one animal welfare experts participated in the workshop, including nine specializing in farm animal welfare (academics = 3, veterinarian = 3, farmer representative = 3). However, the other experts in attendance were general animal welfare experts (academics = 3, vets = 2, industry representative = 1, NGO = 6) therefore were able to competently give an expert opinion on farm welfare issues also. Over the 2 days there was a series of small group (species specific) and large group (to identify cross-cutting issues) exercises and discussions in order to finalize the priority welfare lists for each species group and to rank them.

## Results

### Demographic Information

Experts had a similar age structure across the different species groups and were female-biased, except for small ruminants (Table 1). Experts were predominantly veterinarians or academic researchers (56–68% of experts) with the remainder made up of a range of other areas of expertise, including industry, government, and NGO representation (Table 1). Responses rates were 93, 75, 75, and 93% for round 1, and 93, 75, 81, and 93% for round 2, for pigs, poultry, cattle and small ruminants, respectively.

**Table 1:** Expert demographics, averaged over two rounds of surveys, including age, gender, profession and level of education.

**Variable**	**Cattle**	**Poultry**	**Pigs**	**Small ruminants**
**N**	16	12	15	15
**Age**				
Mean	44	44	42	51
SD	11.2	6.8	10.7	10.5
**Gender**				
Male	6	1	4	8
Female	10	11	11	7
**Profession**				
Researcher	26%	47%	31%	9%
Veterinarian	32%	21%	25%	54%
NGO/charity	13%	21%	13%	16%
Industry Org.	13%	0%	13%	9%
Policy/Gov.	3%	11%	0%	6%
Other	13%	0%	18%	6%
**Education**				
A-levels/highers or below	0%	0%	3%	11%
Bachelor's degree	24%	6%	28%	21%
Master's degree	24%	24%	21%	21%
PhD	48%	64%	41%	32%
Other	4%	6%	7%	15%

### Round 1, Online Surveys

Of the initial list of welfare issues prepared from the literature 35, 37, 42, and 45 issues scored a mean of 3.0 or above for pigs, poultry, cattle and small ruminants, respectively, from Round 1 for either prevalence, severity, or duration of the welfare issue (Tables 2a–d). Standard deviations were relatively high for some issues (Table 2), suggesting there were considerable differences of opinion between experts.

**Table 2:** Ranking of welfare issues from first online survey for prevalence (number of animals affected in the population), severity (degree of individual suffering causes), and duration (period of time for which an individual may experience the welfare issue) for **(a)** pigs, **(b)** poultry, **(c)** cattle, and **(d)** small ruminants.

**Rank order**	**Prevalence**	**Mean rank**	**Severity**	**Mean rank**	**Duration**	**Mean rank**
**(a) PIGS**						
1	Underutilization of NSAIDS	4.92 (0.90)	Delayed euthanasia	4.96 (1.16)	Poor general health status	4.33 (1.37)
2	Lack of veterinary undergraduate training in pigs	4.85 (1.21)	Tail biting outbreaks	4.74 (1.35)	Issues with pet pigs (e.g., lack of pre-purchase research, too easy to buy online, etc.)	4.25 (1.40)
3	Confinement in farrowing crates	4.43 (1.22)	Failure to provide clean/fresh water	4.31 (1.60)	Incorrect selection of animals for the environment	4.18 (1.33)
4	Delayed euthanasia	4.07 (1.54)	Neglect/failure to recognize or treat conditions	4.29 (1.23)	Common pig diseases	4.17 (1.34)
5	Poor biosecurity measures	3.93 (1.44)	Piglet crushing by the sow	4.21 (1.48)	Poor biosecurity measures	4.15 (1.52)
6	High stocking density	3.93 (1.33)	Chronic hunger in sows	4.14 (1.23)	Lack of routine health care	4.14 (1.41)
7	Barren environments	3.93 (1.27)	Inappropriate euthanasia methods	4.05 (1.14)	Too high stocking density	4.14 (1.29)
8	Issues with pet pigs (e.g., lack of pre-purchase research, too easy to buy online, etc.)	3.88 (1.44)	Delayed or inappropriate intervention at farrowing	4.00 (1.41)	Insufficient space allowance	4.07 (1.27)
9	Breeding for larger litters	3.86 (0.95)	Issues with pet pigs (e.g., lack of pre-purchase research, too easy to buy online, etc.)	3.98 (1.34)	Stereotypic or abnormal behavior	4.00 (1.36)
10	Tail biting outbreaks	3.86 (0.95)	Lack of recognition of clinical signs of poor health	3.95 (1.19)	Underutilization of NSAIDS	3.93 (1.35)
11	Common preweaning diseases	3.77 (1.36)	Too high stocking density	3.93 (1.39)	Chronic hunger in sows	3.86 (1.83)
12	Morbidity and mortality of piglets	3.64 (1.15)	Common preweaning diseases	3.92 (0.90)	Lack of veterinary undergraduate training in pigs	3.85 (1.68)
13	Insufficient space allowance	3.64 (1.22)	Behavioral needs not met by environment	3.88 (1.07)	Common preweaning diseases	3.83 (1.40)
14	Mixing and unstable social groups	3.57 (1.16)	High piglet morbidity and mortality	3.79 (1.54)	Poor pasture quality (outdoor pigs)	3.83 (1.40)
15	Common pig diseases	3.46 (1.13)	Lack of shade or shelter (for outdoor pigs)	3.69 1.38)	Poor housing design	3.80 (1.23)
16	Poor general health status	3.43 (1.09)	Poor stockperson handling skills	3.69 (1.32)	Lack of recognition of clinical signs of poor health	3.67 (1.56)
17	Aggression	3.41 (1.21)	Lack of routine veterinary care	3.68 (1.22)	Delayed euthanasia	3.64 (1.50)
18	Pain from management procedures	3.36 (1.34)	Lameness	3.63 (1.40)	Lameness	3.64 (1.15)
19	Chronic hunger in underfed sows	3.36 (1.74)	Poor general health status	3.64 (1.15)	Tail biting outbreaks	3.64 (1.08)
20	Tail docked too short	3.36 (1.01)	Unstable social groups	3.64 (0.93)	Inadequate provision of suitable feed	3.62 (1.16)
21	Poor housing design (flooring, bedding, air quality etc.)	3.33 (1.08)	Inadequate provision of suitable feed	3.63 (1.44)	Behavioral needs not met by environment	3.60 (1.22)
22	Behavioral needs not met by environment	3.31 (1.37)	No hospital pen	3.62 (1.12)	Parasite burdens (outdoor pigs)	3.58 (1.24)
23	Incorrect selection of animals for the environment	3.27 (1.19)	Inability of stockpeople to interpret pig behavior	3.57 (1.09)	Failure to provide clean/fresh water	3.58 (1.51)
24	Abnormal behaviors (stereotypy)	3.25 (1.49)	Common pig diseases	3.54 (1.13)	Breeding for larger litters	3.50 (1.68)
25	Lameness	3.14 (1.03)	Poor quality buildings (resources, air quality etc.)	3.54 (1.05)	Inability of stockpeople to interpret pig behavior	3.50 (1.02)
26	Poor handling skills of stockperson	3.14 0.95)	Predation	3.54 (1.39)	No hospital pen	3.50 (1.38)
27	Pain associated with farrowing	3.08 (1.44)	Poor maternal behavior	3.46 (1.27)	Veterinary “cold spots” for access to pig vets	3.43 (1.76)
28	Predation	3.08 (1.44)	Incorrect selection of animals for the environment	3.45 (1.21)	Poor body condition	3.42 (0.90)
29	Lack of recognition of clinical signs of poor health	3.04 (1.34)	Lack of routine health care	3.43 (1.22)	Lack of shade or shelter (outdoor pigs)	3.38 (1.33)
30	Inability of stockpeople to interpret pig behavior	3.00 (0.78)	Aggression between pen-mates	3.36 (0.84)	Delayed veterinary care	3.34 (0.98)
31	Veterinary “cold spots” for access to pig vets	3.00 (1.47)	Impact of breeding for larger litters	3.36 (1.15)	Poor stockperson handling skills	3.31 (1.18)
32			Pain from management procedures	3.35 (1.60)	High piglet morbidity and mortality	3.14 (1.51)
33			Lack of veterinary undergraduate training in pigs	3.31 (1.38)	Neglect/lack of regular inspection	3.07 (1.33)
34			Confinement (e.g., farrowing crates)	3.21 (1.31)	Aggression between pen-mates	3.07 (0.99)
35			Transport issues	3.16 (1.40)	Method and timing of weaning	3.00 (1.34)
**(b) POULTRY**						
1	Unwanted male chicks	5.43 (1.13)	Outbreak of feather-pecking or cannibalism that is not dealt with swiftly	5.29 (0.95)	Chronic hunger in broiler and turkey breeders	5.25 (1.75)
2	Keel bone fractures and damage	5.14 (0.69)	Poor stockperson handling skills	5.14 (0.90)	Inability to express natural behaviors	5.23 (1.27)
3	Chronic hunger in broiler and turkey breeders	4.88 (2.10)	Chronic hunger in broiler and turkey breeders	5.00 (1.77)	Reduced feather cover	5.00 (0.82)
4	Painful procedures	4.86 (1.35)	Inability to express natural behaviors	4.82 (1.33)	Consequences from breeding decisions (e.g., rapid growth, exaggerated conformation)	5.00 (1.83)
5	Poor handling skills by stockpeople	4.86 (1.60)	Poor foot/leg health	4.78 (1.20)	Artificial lighting regimes	4.88 (1.39)
6	Reduced feather cover	4.71 (0.76)	Consequences from breeding decisions (e.g., rapid growth, exaggerated conformation)	4.72 (1.99)	Inappropriate housing conditions/environment	4.84 (1.48)
7	Consequences from breeding decisions (e.g., rapid growth, exaggerated conformation)	4.67 (2.22)	Hock, breast, and footpad burns from damp litter	4.67 (1.23)	Expression of abnormal behaviors	4.78 (1.13)
8	Inability to express natural behaviors	4.58 (0.89)	Delayed euthanasia of sick/injured birds	4.56 (0.88)	Inadequate housing design	4.48 (1.23)
9	Artificial lighting regimes	4.56 (1.33)	Expression of abnormal behaviors	4.52 (1.04)	Outbreak of feather-pecking or cannibalism that is not dealt with swiftly	4.43 (0.79)
10	Stress associated with transport	4.45 (1.01)	Not seeking or delayed veterinary care	4.50 (1.20)	Incorrect selection of animals for the environment	4.43 (1.27)
11	Poor foot and leg health	4.44 (0.88)	Pain due to management procedures	4.32 (1.64)	Inappropriate diets/nutritional regimes	4.33 (1.60)
12	Lack of appropriate environmental enrichment	4.44 (1.42)	General poor health status	4.25 (1.20)	Hock, breast, and footpad burns from damp litter	4.33 (1.00)
13	Lack of pre-purchase research e.g., buying birds online	4.43 (1.39)	Lack of continuous access to clean/fresh water	4.25 (1.91)	Inadequate social groupings	4.23 (1.64)
14	Hock, breast, and footpad burns from damp litter	4.33 (0.87)	Predation of outdoor birds	4.25 (1.91)	Lack of pre-purchase research e.g., buying birds online	4.14 (1.77)
15	Euthanasia methods	4.15 (1.60)	Keel bone fractures and damage	4.14 (1.22)	Lack of routine healthcare	4.11 (1.27)
16	Abnormal behaviors	4.02 (0.81)	Reduced feather cover	4.14 (1.46)	Poor foot and leg health	3.89 (1.36)
17	Inappropriate housing conditions/environment	3.90 (1.47)	Inappropriate diets/nutritional regimes	4.13 (1.22)	General poor health status	3.83 1.48)
18	Stress and injury from automated handling pre-slaughter	3.86 (1.57)	Inability of stockpeople to recognize clinical signs of poor health	4.13 (1.04)	Keel bone fractures and damage	3.71 (0.76)
19	Lack of poultry specific veterinary undergraduate training	3.86 (1.35)	Physical injury from aggression between birds	4.13 (1.64)	Lack of continuous provision of clean/fresh water	3.63 (1.92)
20	Inadequate housing design (space, air quality etc.)	3.77 (1.33)	Inappropriate housing conditions/environment	3.99 (1.13)	Pain due to management procedures	3.63 (1.21)
21	Uncleanliness of plumage	3.75 (1.49)	Stress associated with transport	3.98 (1.38)	Physical injury from aggression between birds	3.63 (1.60)
22	Delayed removal of dead birds from cages	3.67 (1.51)	Lack of routine healthcare	3.89 (1.05)	Lack of knowledge by caretaker of poultry behavior	3.50 (1.20)
23	Unpreparedness for emergency	3.67 (1.51)	Inadequate housing design	3.86 0.74)	Poor biosecurity	3.50 (1.51)
24	General poor health status	3.52 (1.45)	Unpreparedness for emergency	3.83 (1.72)	Use of “spiking” roosters in flock aggression	3.40 (1.95)
25	Physical injury from aggression	3.50 (1.31)	Inappropriate social groupings	3.79 (0.76)	Build-up of stale/contaminated food	3.38 (1.51)
26	Incorrect selection of animals for the environment	3.43 (1.27)	Use of inappropriate euthanasia methods	3.78 (1.28)	Inability of stockpeople to recognize clinical signs of poor health	3.38 (0.92)
27	Use of “spiking” roosters in flock aggression	3.40 (1.52)	Not using turkey saddles before mating	3.67 (1.53)	Uncleanliness of plumage	3.38 (1.60)
28	Delayed euthanasia of sick/injured birds	3.22 (1.09)	Neglect—lack of regular flock inspections	3.63 (1.60)	Lack of availability of poultry specific medicines	3.25 (0.89)
29	Pain due to management procedures	3.14 (2.04)	Pain caused by turkey semen collection	3.60 (1.52)	Delayed or lack of veterinary care	3.24 (1.40)
30	Severe ascites in broilers	3.14 (1.07)	Poor management of hypothermia	3.43 (1.27)	Lack of poultry specific veterinary undergraduate training	3.14 (1.68)
31	Not seeking or delayed veterinary care	3.13 (1.55)	Artificial lighting regimes	3.33 (1.32)	Delayed euthanasia of sick/injured birds	3.11 (0.60)
32	Lack of knowledge by caretaker of poultry behavior	3.12 (0.99)	Lack of pre-purchase research e.g., buying birds online	3.29 (1.11)	Neglect (lack of regular flock inspection)	3.00 (1.41)
33	Lack of recognition of clinical signs by stockpeople	3.00 (0.76)	Incorrect selection of animals for the environment	3.29 (0.95)		
34	Lack of medicines available for poultry	3.00 (1.31)	Lack of knowledge by caretaker of poultry behavior	3.25 (1.04)		
35	Not using turkey saddles before mating	3.00 (1.41)	Lack of poultry specific veterinary undergraduate training	3.14 (1.46)		
36	Pain caused by turkey semen collection	3.00 (1.16)	Lack of availability of poultry-specific medicines	3.13 (0.99)		
37			Use of 'spiking' roosters in flock aggression	3.00 (0.71)		
**(c) CATTLE**						
1	Early maternal separation (dairy)	5.25 (1.42)	Delayed euthanasia of sick/injured animals	5.25 (0.62)	Poor foot health	4.82 (0.60)
2	Common production diseases	4.67 (0.99)	High calf mortality and morbidity	5.08 (0.79)	Permanent housing (e.g., zero grazing)	4.67 (1.16)
3	High calf mortality and morbidity	4.58 (0.90)	Poor foot health	4.73 (0.91)	Poor general health status	4.58 (1.00)
4	Poor foot health	4.5 (0.93)	Lack of access to veterinarians (e.g., regional “cold spots”)	4.55 (1.29)	Common infectious diseases	4.55 (0.93)
5	Reproductive management practices (e.g., AI)	4.50 (1.45)	Pain caused by calving	4.50 (1.09)	Lack of access to veterinarians (e.g., regional “hot spots”)	4.55 (1.37)
6	Pain caused by routine management practices	4.46 (1.24)	Inadequate water supply	4.42 (1.51)	Lack of proper foot care	4.42 (1.24)
7	Poor colostrum management	4.45 (0.93)	Lack of recognition of clinical signs of ill health by stockpeople	4.33 (0.99)	Consequences from breeding decisions (e.g., exaggerated conformation)	4.36 (1.43)
8	Poor weaning practices (beef)	4.43 (1.35)	Delayed calving intervention	4.27 (0.79)	Poor colostrum management	4.36 (1.21)
9	Underutilization of NSAIDs	4.17 (1.12)	Poor colostrum management	4.27 (1.01)	Lack of ability to perform natural behaviors	4.33 (1.37)
10	Poor health status	4.00 (0.74)	Poor general health status	4.25 (0.97)	Lack of routine health care	4.33 (1.44)
11	Unwanted male dairy calves	3.92 (0.90)	Inappropriate diets/nutritional regime	4.24 (1.45)	Inappropriate housing conditions/environment	4.26 (1.12)
12	Poor cubicle design	3.92 (0.90)	Unskilled service providers (e.g., foot trimmer)	4.18 (0.75)	Common production diseases	4.25 (1.29)
13	Delayed euthanasia of sick/injured animals	3.83 (1.19)	Pain caused by routine management procedures	4.03 (1.40)	High calf mortality and morbidity	4.25 (0.97)
14	Lack of ability to perform natural behaviors	3.83 (1.40)	Lack of routine health care (e.g., vaccinations, etc.)	4.00 (1.35)	Unskilled service providers (e.g., foot trimmer)	4.18 (1.17)
15	Lack of shelter/shade outside	3.75 (0.97)	Lack of proper foot care	4.00 (1.13)	Poor body condition	4.17 (1.20)
16	Transport related issues	3.75 (1.34)	Under-utilization of NSAIDs	4.00 (1.41)	Impact of breeding for increased production	4.08 (1.43)
17	Impact of breeding for exaggerated conformation/defects	3.63 (1.17)	Transport related issues	4.00 (1.47)	Poor lighting regime	4.08 (1.62)
18	Inadequate social groups—under/over-stocking, composition, etc.	3.58 (1.24)	Common infectious diseases	3.84 (1.13)	Mineral deficiency	4.00 (1.10)
19	Common infectious diseases	3.50 (1.17)	Breeding for exaggerated conformation/defects/oversized calves, etc.	3.82 (1.35)	Lack of recognition of clinical signs of ill health by stockperson	3.83 (1.34)
20	Lack of proper foot care	3.50 (1.09)	Use and control of dogs (e.g., biting)	3.82 (1.33)	Inadequate selection of animals for the environment	3.83 (1.47)
21	Intensive finishing systems	3.45(1.13)	Inadequate social groups—under/over-stocking, composition, etc.	3.75 (1.07)	Inadequate water supply	3.75 (1.22)
22	Inappropriate housing conditions/environment	3.42 (0.79)	Permanent housing (e.g., zero-grazing)	3.75 (1.55)	Inappropriate diets/nutritional regime	3.61 (1.49)
23	Poor stockperson handling skills	3.42 (0.78)	Early weaning practices	3.75 (1.83)	Abnormal behaviors	3.58 (1.17)
24	Insufficient biosecurity measures	3.33 (1.23)	Exposure to toxic plants	3.67 (1.30)	Lack of knowledge by caretaker of cattle behavior	3.58 (1.56)
25	Poor air quality	3.33 (0.49)	Inappropriate housing conditions/environment	3.67 (1.16)	Poor calf management	3.57 (1.40)
26	Lack of recognition of clinical signs of ill health by stockperson	3.33 (0.78)	Lack of shelter/shade outside	3.67 (0.99)	Inadequate social groups—under/over-stocking, composition, etc.	3.55 (1.56)
27	Poor body condition	3.33 (0.99)	No hospital pens	3.67 (1.61)	Lack of biosecurity measures	3.50 (1.38)
28	Limited key resources (e.g., cubicles, feeding spaces)	3.33 (0.65)	Consequences from breeding decisions (e.g., rapid growth, exaggerated conformation)	3.64 (0.92)	Build-up of stale/contaminated feed	3.45 (1.44)
29	Permanent housing (zero-grazing)	3.25 (0.87)	Poor maternal behavior	3.64 (1.43)	Neglect (failure to regularly inspect)	3.36 (1.12)
30	Poor pasture quality	3.25 (0.87)	Unpreparedness for mass depopulation	3.63 (1.60)	Consequences of the presence of horns	3.33 (1.72)
31	Poor calf management	3.18 (1.08)	Poor stockperson handling skills	3.58 (1.00)	Poor stockperson handling skills	3.25 (1.06)
32	Lack of knowledge by caretaker of cattle behavior (ethology)	3.17 (1.03)	Inappropriate drying off techniques	3.55 (1.13)	Lack of shelter/shade outside	3.17 (1.12)
33	Poor maternal behavior	3.09 (1.51)	Neglect—failure to inspect regularly	3.55 (0.93)	Aggression between cattle	3.08 (1.00)
34	Inappropriate diets/nutritional regime	3.08 (1.00)	Lack of knowledge by caretaker of cattle behavior	3.50 (1.38)	Exposure to toxic plants	3.00 (1.13)
35	Insufficient space allowance	3.08 (0.90)	Presence of horns	3.50 (1.51)	Pain caused my management practices	3.00 (1.04)
36	Build-up of stale/contaminated feed	3.00 (1.34)	Failure to select animals for the environment	3.42 (1.00)	Underutilization of NSAIDs	3.00 (1.04)
37	Poor maintenance of equipment, facilities etc.	3.00 (0.85)	Unwanted male dairy calves	3.33 (1.23)	Intensive finishing systems	3.00 (1.10)
38			Aggression between cattle	3.25 (1.06)		
38			Poor body condition	3.17 (1.12)		
39			Impact of build-up of stale/contaminated feed	3.09 (1.14)		
40			Lack of biosecurity	3.08 (1.00)		
41			Abnormal behaviors	3.00 (0.85)		
42			Inability to perform natural behaviors	3.00 (1.13)		
**(d) Small ruminants**						
1	Lack of suitable analgesics	4.74 (1.18)	Delayed euthanasia of sick/injured animals	5.08 (1.17)	Breeding for exaggerated conformation/rapid growth	4.52 (1.34)
2	Lameness	4.69 (1.11)	Lameness	5.00 (0.82)	Lameness	4.46 (0.78)
3	Biosecurity issues	4.58 (1.31)	Lack of suitable analgesics	4.50 (1.24)	Tails docked too short	4.45 (1.75)
4	Not seeking, or delayed, veterinary care	4.33 (0.99)	Not seeking, or delayed, veterinary care	4.42 (1.08)	Delayed euthanasia of sick/injured animals	4.25 (1.29)
5	Gastro-intestinal (GI) parasites	4.31 (1.03)	Common health issues (e.g., myiasis)	4.38 (1.39)	Lack of veterinary undergraduate training in small ruminants	4.09 (1.22)
6	Lack of veterinary undergraduate training in small ruminants	4.00 (1.35)	Inability of stockpeople to recognize clinical signs of ill health	4.33 (1.30)	Impact of mineral deficiency	4.09 (0.83)
7	Delayed euthanasia of sick/injured animals	3.92 (1.24)	Exposure to and ingestion of toxic plants	4.31 (1.49)	Inadequate provision of feed/forage	4.09 (1.38)
8	Pain due to management procedures	3.87 (1.12)	High neonatal morbidity and mortality	4.22 (1.09)	Incorrect selection of appropriate breed for environment	4.09 (1.70)
9	Permanent housing of dairy goats	3.67 (1.44)	Stress caused by social isolation	4.17 (1.64)	GI parasites	4.00 (0.91)
10	Unwanted male goat kids	3.67 (1.30)	Lack of shade or shelter	4.15 (1.35)	Lack of access to veterinary care	4.00 (1.53)
11	Common sheep/goat health issues	3.62 (1.39)	Pain associated with lambing	4.13 (1.24)	Not seeking, or delayed, veterinary care	4.00 (1.04)
12	Lack of routine health care	3.62 (1.26)	Use and control of farm dogs (e.g., biting)	4.09 (1.14)	Permanent housing of dairy animals	4.00 (1.58)
13	Poor health status	3.54 (1.20)	GI parasites	4.00 (0.82)	Poor pasture management	3.92 (1.38)
14	Lack of shade or shelter	3.46 (1.27)	Poor health status	4.00 (1.08)	Lack of routine health care	3.85 (1.21)
15	Impact of mineral deficiency	3.42 (1.08)	Predation	4.00 (1.33)	Biosecurity issues	3.83 (1.53)
16	High neonatal mortality and morbidity	3.39 (1.06)	Pain due to management procedures	3.94 (1.38)	Common sheep/goat health issues	3.77 (1.09)
17	Inadequate maintenance of buildings and facilities	3.36 (0.93)	Poor pasture management	3.92 (1.24)	Poor body condition	3.77 (1.01)
18	Poor pasture management	3.33 (0.78)	Unskilled/Incompetent service providers	3.90 (1.52)	Poor dental health/tooth loss	3.77 (1.01)
19	Lack of access to vet care (regional “cold spots”)	3.31 (1.32)	Lack of routine health care	3.85 (1.14)	Poor health status	3.77 (1.01)
20	Poor dental health/tooth loss	3.31 (0.86)	Inadequate provision of feed/forage	3.83 (1.47)	High stocking density of housed animals	3.69 (1.11)
21	Inadequate feeding for animal requirements	3.25 (0.62)	Lack of veterinary undergraduate training in small ruminants	3.83 (1.12)	Lack of shade or shelter	3.69 (1.49)
22	High stocking density of housed animals	3.23 (1.09)	Breeding for exaggerated conformation/rapid growth	3.82 (1.25)	Inability of stockpeople to recognize clinical signs of ill health	3.67 (1.23)
23	Poor handling practices (e.g., dragging by fleece)	3.21 (1.09)	Inadequate management of orphan lambs/kids	3.75 (1.31)	High neonatal morbidity and mortality	3.59 (1.31)
24	Incorrect selection of appropriate breed for the environment	3.18 (1.33)	Impact of mineral deficiency	3.75 (0.87)	Lack of suitable analgesics	3.59 (1.56)
25	Transport issues	3.18 (1.29)	Neglect (failure to regularly inspect animals)	3.75 (1.55)	Inadequate maintenance of buildings and facilities	3.55 (1.37)
26	Breeding for exaggerated conformation/defects	3.12 (1.35)	No hospital pens	3.75 (1.71)	Inadequate management of orphan lambs/kids	3.50 (1.09)
27	Inability of stockpeople to recognize clinical signs of ill health	3.08 (0.67)	Biosecurity issues	3.67 (1.30)	Poor housing design (e.g., flooring, ventilation)	3.46 (1.42)
28	Neglect (failure to regularly check animals)	3.00 (1.13)	Horn-related injuries	3.67 (1.61)	Unwanted male goat kids	3.45 (1.64)
29	No hospital pens	3.00 (0.95)	Incorrect selection of appropriate breed for the environment	3.67 (1.29)	Fleece cleanliness	3.40 (1.51)
30	Tails docked too short	3.00 (0.89)	High stocking density of housed animals	3.62 (1.19)	Neglect (failure to regularly check animals)	3.36 (1.21)
31			Inability of stockpeople to interpret behavior	3.54 (1.3)	Inability of stockpeople to interpret behavior	3.31 (1.25)
32			Common infectious diseases	3.46 (1.39)	No hospital pens	3.25 (1.22)
33			Lack of access to veterinary care	3.46 (1.39)	Common infectious diseases	3.23 (1.17)
34			Poor handling practices	3.46 (1.38)	Horn-related injuries	3.18 (1.25)
35			Restraint of foster ewes	3.45 (1.51)	Inadequate provision of clean/fresh water	3.18 (1.33)
36			Inadquate provision of clean/fresh water	3.42 (1.31)	Use of and control of farm dogs	3.18 (1.08)
37			Stress associated with transport issues	3.39 (1.29)	Inappropriate shearing practice (e.g., winter shearing, failing to shear)	3.15 (1.56)
38			Poor body condition	3.38 (0.87)	Stress caused by social isolation	3.08 (1.44)
38			Inadequate maintenance of buildings and facilities	3.36 (1.36)	Poor handling practices (e.g., dragging by fleece)	3.00 (1.28)
39			Poor housing design	3.28 (1.43)		
40			Tail docked too short	3.27 (1.01)		
41			Poor dental health/tooth loss	3.23 (0.83)		
42			Unwanted male goat kids	3.17 (1.40)		
43			Social behavior issues (e.g., mixing)	3.09 (1.22)		
44			Inappropriate shearing practice (e.g., winter shearing)	3.00 (1.20)		
45			Permanent housing of dairy goats	3.00 (1.04)		

*Values are mean scores (with S.D) of experts opinion, derived from a Likert score from 1 (low) to 6 (high), for all issues that scored at least 3.0 (and therefore considered by experts to be at least somewhat important)*.

### Round 2, Online Surveys

The level of agreement between experts on the average ranking for each welfare issue was relatively low (Fleiss' κ, Table 3), with particularly low levels of agreement between cattle experts. For pigs 86% of experts agreed that delayed euthanasia and tail biting were the most severe challenges to welfare, but did not agree on other high scoring issues. For poultry good agreement was achieved for the prevalence of keel bone fractures (75%), the severity of poor foot and leg health (78%), and duration of impact of artificial lighting regimes and inappropriate housing conditions (both 75%). Cattle experts had good agreement (77–92% of experts agreed on the ranking position) on the issues ranked most highly for prevalence, severity and duration (poor foot health, high calf mortality, common production diseases, poor general health, and delayed euthanasia), but agreement elsewhere was poor. For small ruminants, only the severity of lameness (86%) and lack of suitable analgesics (79%) had high agreement amongst the most highly ranked welfare issues.

**Table 3:** Fleiss' kappa (κ) for agreement between experts in the ranking of welfare issues based on prevalence, severity, and duration for cattle, pigs, poultry, and small ruminants.

**Species**	**Prevalence**	**Severity**	**Duration**
Cattle	0.159	0.255	0.176
Pigs	0.229	0.228	0.241
Poultry	0.296	0.336	0.297
Small ruminants	0.318	0.325	0.338

### Round 3, Workshop

Final rankings of welfare issues for each species were achieved during the workshop (Table 4). During the course of the Workshop experts considered the severity and duration of welfare issues together (considering the welfare of individual animals), and prevalence of a welfare issue was considered alone (considering the average welfare impact on a population of animals). The experts were asked to create a list of approximately 10 welfare issues; however, this was not strictly upheld and the lists contain between 8 and 11 issues.

**Table 4:** Ranked welfare priority issues of farmed animals determined using a modified Delphi method for pigs, poultry, sheep and beef cattle and dairy cattle and dairy goats, for individual animals (considering severity and duration) and for the population (prevalence).

**Species**	**Ranking**	**Priority welfare issues**	
		**Prevalence**	**Severity × duration**
Pigs	1	Pain from management procedures	Behavioral needs not met
	2	Tail biting	Tail-biting
	3	Behavioral needs not met	Inadequate stockperson skills
	4	Poor housing design (floor, ventilation, maintenance, layout)	Delayed euthanasia
	5	Poor general health status	Lameness
	6	Inadequate stockperson skills	Poor general health status
	7	Lameness	Breeding for large litters
	8	Gastric ulcers and inadequate feeding	Inadequate/unsuitable feed
	9	Aggression	Aggression
	10		Riding behavior
	11		Lack of use of analgesics
Poultry	1	Consequences from breeding decisions	Consequences from breeding decisions
	2	Inappropriate housing conditions/environment Inability to express natural behaviors (abnormal behaviors)	Inappropriate housing conditions/environment Inappropriate social grouping
	3	Transportation and handling issues	Inability to express natural behaviors (abnormal behaviors)
	4	Painful/uncomfortable conditions due to management/housing Lack of knowledge by caretaker of poultry behavior	Neglect
	5		Lack of knowledge of poultry behavior
	6	Painful procedures	Transport related issues
	7	Delayed euthanasia	Unpreparedness for emergency
	8	Physical injury from aggression	Euthanasia methods
Sheep + beef cows	1	Lack of perception of painful conditions and pain management. Lack of recognition of underlying poor health status (i.e., not just thin animal).	Neglect
	2		Lameness
	3	Lack of local veterinary care Lack of staff to quickly deal with health issue	Sheep scab Mastitis
	4	High neonatal morbidity and mortality Lameness Chronic GI parasites Sheep scab	Dystocia
	5		Inappropriate nutrition
	6		Overstocking/stocking density in housed animals
	7		
	8	Predation/worrying (wildlife and dog attacks)	
	9	Poor dental health	
	10	Lack of appropriately trained staff/contractors (e.g., shearers, transporters)	
Dairy cows + dairy goats	1	Neonatal morbidity and mortality	Inappropriate nutrition
	2	Poor pain management	Neonatal morbidity and mortality
	3	Inappropriate nutrition	Poor stockmanship skills
	4	Production diseases e.g., lameness	Social behavior issues (e.g., mixing animals, aggression, etc.)
	5	Poor stockmanship skills	Poor pain management
	6	Social behavior issues (e.g., mixing animals, aggression, etc.)	Infectious diseases
	7	Infectious diseases	Euthanasia techniques—specifically for killing goat kids
	8	Lack of opportunity to display species specific behaviors (goats e.g., browsing/climbing)	
	9	Euthanasia techniques—specifically for killing goat kids	

*Issues in the same box have equal ranking*.

During the workshop discussion process the cattle and small ruminant experts decided to combine the welfare issues faced by dairy goats and dairy cattle, and also sheep and beef cattle as the issues facing each group were more similar. Therefore, the results from the workshop have been presented in this manner.

## Discussion

The study was able to achieve consensus among experts of what was considered the most important welfare issues for farmed species, with the workshop outcomes including all the highly ranked issues from the first online survey, which had good agreement in the second. However, the opportunity to discuss each issue in more detail, and in some cases to combine a number of issues into a single category (for example, combining different aspects of housing design or diseases relating to production), meant that experts had better agreement and reported more confidence in the final rankings produced in the workshop. Overall, there were a number of common issues that arose in all or nearly all species including: inadequate or inappropriate nutrition; inability of stockpeople to recognize and/or treat welfare issues (such as pain or behavioral problems); foot and leg health resulting in lameness; chronic or endemic health issues; euthanasia delay and methods (particularly those used on farm for killing surplus or unwanted male animals), and neonatal mortality and morbidity. A number of specific welfare issues were identified including abnormal or damaging behaviors in pigs, poultry, and dairy animals; inadequate or poor housing and environments for pigs and poultry; consequence of breeding decisions and genetic selection strategies in pigs and poultry; lack of access to veterinary care for beef and sheep, and issues with handling and transport in sheep, beef and poultry.

The study focused on welfare issues experienced by UK farmed livestock, and the experts who took part in the study were based in the UK. However, similar farming systems and methods are used across Europe and other industrialized countries and thus the outcomes are likely to be relevant to the livestock production of other countries with similar systems to those used in UK. A proviso might be that some management and husbandry practices are not legally permitted in the UK that may be allowed elsewhere (e.g., battery cages and gestation stalls are banned within the EU), and some practices are permitted in UK that may be illegal elsewhere (e.g., tail docking of piglets and lambs). Thus, consideration of these issues may be more or less relevant in some countries.

### Common Issues Across Farmed Species

#### Inappropriate Nutrition

Inappropriate nutrition was identified as a source of welfare concern for individual animals (as indicated by the severity and duration of suffering), and also as perceived to be prevalent or widespread for dairy animals and pigs. This reflected a broad category of concern related to high metabolic rate and consequences for feeding dairy cows (14), low body condition score in sheep and beef (15, 16), and methods of feeding in pigs, which may lead to gastric ulceration (17). These concerns were chiefly those experienced by the breeding stock, which may remain on the farm for several years, rather than the young animals destined for prime meat production. However, this does expose that feeding and nutritional management of pregnant and breeding animals may be sub-optimal, either through deliberate food restriction [e.g., of broiler breeders and pregnant sows; (18)] or through difficulties in managing forage resources at certain times of the year (19, 20).

#### Lack of Stockperson Skills or Knowledge

For all species lack of knowledge, training, skills, or expertise in animal behavior and welfare, and/or access to knowledgeable veterinary care, were highlighted as both a source of individual welfare issues in all species, and as prevalent as a welfare issue within each industry, in the experts' opinion. This may be because behavior, pain, and welfare issues are not routinely included in training for stockpeople or livestock advisors, and farm animal behavior and welfare teaching is often limited in veterinary education. Delayed access to veterinary care may be due to economic concerns (veterinary care of individual animals may be considered economically unviable), an inability to provide individualized care to extensively kept species or poultry (leading to individual neglect where pain or disease may go undiagnosed or untreated), incorrect diagnosis and inappropriate therapies from farmers or lack of access to specialist vets in isolated areas. Veterinary care is rarely sought by sheep/goat farmers (21), and Lovatt (22) suggests that the veterinary services available for sheep are generally the general farm veterinary practitioner, and not a specialist (unlike the pig and poultry sector). However, veterinarians can have the biggest influence on farmer behavior [e.g., in uptake of strategies to reduce pig aggression (23)]. Therefore, improved access or use of veterinary advice for behavior or welfare problems would have benefit in improving animal welfare. These are complex issues but may be partially addressed through improved staff training programmes, consideration of veterinary Continuing Professional Development (CPD) in these specific areas and better on-farm protocols.

#### Pain Recognition and Management

A prominent issue in the area of caretaker knowledge, as identified by the experts, concerns the lack of pain recognition, and pain management. The source of the pain can be from a variety of common diseases, physical trauma, and through the imposition of painful management procedures. Stockpeople may not recognize the subtle behavioral indicators of pain [e.g., as found by (24)], and pain following management procedures may be unobserved or considered relatively trivial, despite considerable research suggesting otherwise [e.g., (25, 26)]. In some instances, stockpeople may recognize that an animal is in pain, but lack access to suitable medication to administer, feel unable to deal with the issues [as shown by O'Kane et al. (27)] or have concerns regarding residues. This is particularly problematic for small ruminants, where few analgesic and anesthesia options are available (28), which can limit the ability of veterinarians to effectively manage animal pain. This is an obvious area of concern that requires further research or legislation.

#### Lameness and Mobility

Lameness and mobility issues were of concern for all species, arising from selection for rapid growth in pigs and broilers, footpad dermatitis in poultry, infectious agents in sheep and poor claw health in cattle. A number of studies have provided good evidence for the suffering caused by these issues (29–32), the impact on production losses (33–35) and have defined the prevalence of these conditions in the UK. For example, a study of 80 farms over 2.5 years suggested dairy cow lameness varied from 16.2 to 19.3% over the year (36). A more recent study by Vee Randall et al. (37) reported that across 43 farms lameness was 30.1% (7.3–60.6%) from a total of 5,620 dairy cows. In broiler chickens 27.6% of 40 weeks old birds were lame (38), despite on farm culling for poor leg health. Lameness has also been reported to affect nearly 5% of sheep in England (39) and 4.5% of sows (40). Given the extensive knowledge about the impact and treatment of lameness in different species, development of effective education, training and intervention programmes are required to reduce this area of farm animal welfare concern.

#### Chronic and Endemic Health Issues

Experts raised the issue of untreated or unresolved chronic disease issues, and poor health issues, across all the farmed mammalian species. Some of these were specifically linked by experts to production, such as mastitis in dairy animals, lameness or dystocia, whereas others may reflect housing or hygiene practices, failure to maintain biosecurity and the presence of endemic disease. Production diseases are considered those that arise through a complex interaction of pathogen presence, with contributory factors such as housing, feeding or management that facilitate disease spread or persistence. In addition to causing sickness, pain and malaise, poor animal health can lead to an increase in the use of antibiotics on farm, and limit animal production [as described for Johnes disease: (41, 42)]. Although larger and more intensive farms may be better at maintaining biosecurity than smaller or more extensive farms, intensification and crowding can increase stress and disease pressure. Consumers generally are more supportive of changes to housing and management that reduce intensification as a way of managing disease, compared to other approaches, and are particularly concerned about antibiotic use, human and animal health and food safety in these systems (43). In addition to various disease control strategies, such as quarantine, vaccination or testing and culling, McAloon et al. (44) has highlighted improved communication with farmers about best practice to limit disease transmission as a requirement to deal with these issues.

#### Delayed Euthanasia

Delayed euthanasia, i.e., not killing animals that are suffering in a timely manner, and the methods used to kill animals, particularly those for unwanted male animals in the dairy and egg industries were considered a significant source of individual suffering (delayed euthanasia) and perceived to be prevalent (methods of killing). Delayed euthanasia can occur for several reasons, including extending an animal's time for recovery from an illness or injury “to give them a chance,” inexperience of the stockperson in either assessing an animal's prognosis or carrying out the procedure of euthanasia, or waiting for the animal to complete a stage of production before finally being euthanized. For many good stockpeople who care for the animals in their charge it can be a very difficult decision to euthanize an animal. Blackwell (45) has found that it was easier for farm stockpeople to euthanize a sick or injured pig if the farm had a written policy that clearly stated the conditions when an animal should be euthanized. Euthanasia protocols and sufficient staff training in procedures provide staff with reassurance and can reduce the suffering of animals. A particular issue for the dairy and poultry industries is dealing with unwanted or surplus male animals, and this was highlighted for dairy goat kids in particular. As low value animals in a production system that requires female animals, male goat kids are sometimes killed on farm by a variety of methods that include manual killing. However, these methods may have low reproducibility and can impact on welfare, thus methods such as use of non-penetrating percussive devices may improve kid euthanasia practices on farm (46).

#### Neonatal Mortality and Morbidity

Mortality and morbidity of young mammalian livestock was ranked highly in ruminant livestock, for both the impact on individual suffering and prevalence, and as a consequence of breeding for larger litters in pigs [as discussed by Rutherford et al. (47)]. Concern for the early separation of young livestock from their mothers, particularly in dairy animals and poultry, but also early weaning in piglets, additionally contributed to the priority given to this issue. High mortality can also be considered as a production disease, as described above, with factors such as housing and repeat breeding affecting piglet survival (48), nutrition, hygiene, and litter size contributing to lamb mortality (49) and genetics influencing calf mortality (50). In addition, the failure of passive transfer of immunity, particularly in dairy calves, is a significant risk factor for poor neonatal outcomes. As with other health issues, these problems are complex and multi-factorial, but there has been a significant body of work to improve rates of neonatal mortality in different farmed species. Opportunities to implement these changes would require improved communication to farmers, with solutions tailored to farm characteristics (49), where veterinarians and others can play a role.

### Species-Specific Welfare Issues

#### Housing and Environment

For farmed species that are often managed intensively (pigs and poultry), inadequacy of the housing environment and an inability of the management system to meet behavioral needs, leading to damaging behaviors such as tail-biting or feather-pecking, were amongst the most important concerns, as rated by the experts. In modern commercial production systems, pigs and poultry are often confined within simple, invariant, housing systems that offer little potential to accommodate their highly motivated species-specific behaviors (51, 52). There is substantial evidence that animals suffer when they are unable to show motivated behaviors. For example, when sows are prevented from expressing nest-building behaviors by the restrictive nature of the farrowing crate (53), and insufficient substrate is given for pigs to show rooting behavior (54). In poultry, chickens may be unable to show dust-bathing or nesting behavior (52), because the housing does not provide sufficient space or a suitable substrate. Under UK legislation, pigs must be provided with environmental enrichment, and laying birds and pullets must be housed in “enriched” cages, as barren “battery” cages have been banned in the EU since 2012. Enriched cages do provide hens the opportunity to nest, perch and dustbathe, however, the provisions of these resources can often fall short of ideal and still supress the ability of birds to express some natural behaviors. In addition, often only a minimal amount of environmental enrichment is provided for pigs which, although it might provide some welfare improvement (55), falls well short of truly meeting the animals behavioral needs. A consequence of this is that tail-biting, when pigs bite and chew the tails of pen-mates (56), remains a significant welfare problem, which is often dealt with by docking tails to limit the ability of pigs to carry out this damaging behavior. Although tail-docking is regulated in the EU and should not be a routinely performed procedure, it is still carried out on 80% of farms in UK (57) and occurs without provision of analgesia or anesthesia. Tail-docking as a welfare solution to tail-biting, therefore, involves the acute pain associated with docking, and does not address the underlying causes of tail-biting. As with many of the welfare issues for farmed animals identified and prioritized in this study, tail-biting is influenced by a number of factors, including barren housing, suboptimum nutrition (including misdirected foraging, lack of fiber, restricted feeding regimes), poor health, environmental stressors, stocking density and group size (58). Thus, it can be a difficult problem to mitigate, although recent studies have demonstrated that pigs can be successfully managed with long tails by environmental improvements (59, 60) although these may be financially less profitable than tail-docking (57).

#### Breeding and Genetic Issues

The most important welfare issue for poultry, at both the individual and the population level, was considered to be the negative consequences that can arise from breeding decisions. In broiler chickens, selection for rapid growth over the last 50 years has had a dramatic impact on the time taken for birds to reach slaughter weight (e.g., increased growth rates from 25 to 100 g per day), has led to exaggerated conformation through selection for breast muscle and has enormously improved feed conversion rates. The consequences of this rapid growth has led to a number of welfare issues including a significant increase in leg weakness and an inability to walk, cardiovascular disease, musculo-skeletal disorders and reproductive problems in the parent birds unless subjected to severe food restriction (18, 61). For example in a survey of broiler leg health, nearly a third of birds showed poor locomotion, and 3.3% were non-ambulatory (38). Lame birds will preferentially select a feed containing an analgesic (62), which then improved their walking ability, suggesting that altered gait was associated with pain. Poor leg health also contributes to other welfare issues, such as breast blisters, as the lame birds spend most of the time lying, which exposes them to contaminated litter for long periods of time. The welfare issues experienced by broiler chickens have been shown to be related to increased growth rate (38), which, although partly achieved through changes in management practice, has been largely due to genetic selection (63, 64). A number of producers are now acknowledging this issue, but alterations in breeding goals to include aspects of welfare would be required to deal with this issue (63).

Concerns were also raised for the welfare consequences of genetic selection of pigs, specifically selection for larger litter sizes. In UK and Denmark, litter sizes have increased from an average of 11.7 and 12.1 piglets per litter in 1996 to 12.0 and 16.6 piglets per litter in 2011, respectively (47). This has led to a parallel increase in prenatal and pre-weaning mortality, from 7.1–7.4% to 17.9–18.2%, respectively, to 10.8 and 23.5% in 2011. Piglet morbidity and mortality is a significant concern for these larger litters, as well as the associated management practices to deal with large numbers of piglets, as the number of viable piglets may exceed the number of available teats (65). These can include use of nurse-sow systems, early and split weaning and use of artificial rearing systems. These systems impose welfare challenges on the sow, such as prolonged confinement during lactation, as well as on the piglet. Although not included in the final lists of most important welfare issues for other species, the consequences of breeding decisions also featured in the important welfare concerns for all species. Because of their long term (often whole life) and enduring impact on animal welfare, these are significant welfare costs, which require longer term approaches to designing appropriate breeding goals (64) to be mitigated.

#### Handling and Transport

Welfare issues associated with animal handling and transport were highlighted particularly for poultry, and for extensively managed animals (sheep and beef) with little experience of human contact before handling. In poultry, different risk factors are important depending on the type of bird in transit, for example the risk of bone breakages is high in end-of-lay hens, which require gently handling and catching before transport. In general, however, the collection, handling and loading of poultry for transport is stressful for birds [e.g., (66–68)]. Ventilation and environmental control are also very important for transport of poultry, which have a relatively narrow thermoneutral range and are susceptible to high and low temperatures in transit (which can occur simultaneously in the same load at different locations). Under EU directives (EC1/2005), poultry can be transported for up to 12 h without provision of feed and water. This also acts as an effective limit on duration of transport as providing food and water in transit for poultry is largely impossible with current transport methods. Petracci et al. (69) suggest an estimate of 0.35% mortality in broilers in transit, more recent studies suggest broiler mortality ranges from 0.1 to 0.6% and a higher mortality is seen in end-of-lay hens (0.4–1.0%) compared to meat chickens (70). Risk factors for increased mortality were length of journeys, stocking density in transit and ambient temperature, although farm factors, such as catching practices are also important [reviewed by (70, 71)]. Thus, despite some regulation of animal transport, welfare was still considered an issue for animals in transit, and implementation of best practice (e.g., the EU Animal Transport Guides: Consortium of the Animal Transport Guides Project (2017) “Good practices for animal transport in the EU”: http://animaltransportguides.eu/) may help to reduce the welfare costs of transport to slaughter.

### Limitations of the Approach

Although Delphi studies are popular and can address problems that would otherwise be contentious or intractable, it is important to acknowledge their limitations. Outcomes from a Delphi are based on the agreement between experts, rather than empirical objective data. However, this can be valuable in scoping the issues when these data are not readily available. The number of experts in this study was relatively small [this was close to the numbers recommended in some papers e.g., (72) but lower than others e.g., (73)], although these were drawn from a larger pool of 145 experts, and ideally the reliability of the study should be tested with other groups of experts. There is no statistical test for reliability in Delphi studies. However, the outcomes of this study met with the suggested criteria for credibility (54) as participants were interested and knowledgeable about the field, reasoned discussion and debate was part of the process in the workshop and supporting data from the literature have been presented here, supporting the validity of the outcomes. A previous (anonymous) study of farm animal welfare using a Delphi approach concluded that problems arise when animals are kept in environments that do not meet behavioral needs (74). This was broadly corroborated in our paper, particularly for pigs, poultry, and dairy. In UK, sheep and beef cattle are usually managed extensively, and their environments are more likely to meet behavioral needs, and thus the prioritized welfare issues focused on issues with the treatment of disease and injury. We took a broad approach to welfare issues and included both risk factors (such as housing or management practices) as well as welfare outcomes (such as tail-biting or diseases). In practice this was sometimes problematic for the experts and did require them to consider the importance of issues that were not necessarily on the same scale. In future work separation of these issues may be beneficial.

The Delphi method does not allow us to analyze why particular issues were prioritized and to explore the rationale behind some issues being prioritized over others. Welfare is often seen as being related to biological functioning of the animal, the naturalness of the environment and/or the feelings or subjective experiences of the animal (3). However, a particular preference for or bias toward only one of these areas can lead to different decisions about welfare. The Delphi method attempts to remove these potential differences of opinion by seeking an overall consensus, which may involve weighing the relative importance of these arguments. This may explain the relatively low agreement with the online surveys, where discussion was not possible, and the improvement in agreement in the workshop resulting from discussion. We cannot, however, rule out the possibility that the workshop, by not being anonymous, may have allowed particularly dominant individuals to influence the apparent consensus.

## Conclusions

Overall, the modified Delphi methodology was effective in allowing animal welfare experts to reach consensus on the current most important welfare issues for farmed animals. There is always the possibility that different results might ensue depending on the panel of experts participating (75). However, in studies such as this one, where empirical evidence is unavailable, the Delphi method does provide a framework with which to work. Also, by inviting stakeholders from a range of professional and academic disciplines to participate, there is more likely to be a balance of inevitable discipline-specific biases (9).

The phrasing of survey questions, and focus of the study, was on “welfare issues,” which means that the study only considered negative aspects of welfare. However, as outlined earlier, positive animal welfare is becoming more important and was not addressed in this study, but could be included in further work to provide a more complete view of the welfare of farmed animals. The final priority welfare issues contained a mix of animal-, resource-, and management-based factors, which can be addressed by a mixture of further research, education, communication, and policy-change strategies to implement existing knowledge, or to understand the issues and how to mitigate them in more detail, and to achieve an improvement in the welfare of farmed animals.

## Data Availability Statement

The datasets generated for this study are available on request to the corresponding author.

## Ethics Statement

The studies involving human participants were reviewed and approved by Human Ethical review Committee (HERC), Royal (Dick) School of Veterinary Studies, University of Edinburgh. The patients/participants provided their written informed consent to participate in this study.

## Author Contributions

CD designed the project and obtained funding with the assistance of HB, MC, and AL. FR-L was employed to work on the project and conducted the work under supervision of CD, MC, and HB, and produced the first draft of the manuscript. CD, HB, MC, and AL edited and completed the final draft of the paper.

### Conflict of Interest

The authors declare that the research was conducted in the absence of any commercial or financial relationships that could be construed as a potential conflict of interest.
