# Supplementary material for: Prioritization of Farm Animal Welfare Issues Using Expert Consensus
Source: Front Vet Sci. 2020 Jan 10;6:495. doi: 10.3389/fvets.2019.00495 (PMC6967597; doi:10.3389/fvets.2019.00495)
Supplement: Supplementary file 1 [file Table_1.DOCX]

**Supplementary Material - Original Animal Welfare Lists**

Original list of welfare issues used in first surveys (not ranked) as derived from the literature / other resources

*Pig*

(n=80)

1. Lack of routine health care (e.g. vaccinations, parasite prevention, etc)
2. Poor general health status (e.g. metabolic/reproductive/respiratory disease)
3. Lack of recognition of clinical signs of poor health/disease (by stockperson)
4. Common swine diseases (e.g. Brucellosis, PED, E.coli, etc)
5. Common pre-weaning diseases (e.g. PRRS, Rotavirus, etc)
6. Poor hoof health / lameness
7. Failure to recognize or treat painful conditions (e.g. lameness, arthritis, etc)
8. Underutilisation of NSAIDS
9. Delayed veterinary care
10. “Cold spots” in UK of access to swine veterinarians
11. Excessive parasitic burdens (outdoor pigs)
12. Poor body condition (thin or obese)
13. High morbidity/mortality rates of piglets
14. Method and timing of weaning
15. Poor pest control / bio-security (e.g. birds, rodents, insects)
16. No provision of hospital pen for sick animals
17. No provision of relief pen for sows not coping in group housing systems
18. Inadequate provision of feed/forage (quality or quantity)
19. Non-continuous provision of clean water
20. Feeding a diet appropriate for animal age/stage of production (e.g. parturient)
21. Chronic hunger in sows (fed limited ration)
22. Build-up of stale/contaminated feed/water
23. Insufficient space allowance: no opportunity to exercise / avoid aggressive interactions
24. Insufficient space allowance: no opportunity to separate feeding, lying and dunging areas
25. Unstable social groups (mixing unfamiliar pigs disrupts hierarchy – aggression)
26. Confinement systems e.g. farrowing crates
27. High stocking densities
28. Stress from solitary living (temporary of permanent)
29. Pain caused by castration
30. Pain caused by tail docking
31. Pain and/or distress caused by ear notching
32. Pain and/or distress caused by tattoo slapping
33. Pain and/or distress caused by teeth clipping (piglets)
34. Pain and/or distress caused by tusk trimming (boars)
35. Pain and/or distress caused by nose rings (boars)
36. Poor maternal behaviour (sow-piglet bonding)
37. Belly-nosing (re-directed suckling behaviour towards litter mates)
38. Presence of abnormal behaviours (stereotypy, bar biting, sham chewing)
39. Tail biting outbreaks
40. Aggression between pen-mates
41. Lack of provision, or poor quality, bedding in pens
42. Poor flooring surface / hygiene (e.g. wet, soiled lying areas, slippy)
43. Poor quality of buildings / equipment (e.g. handling facilities) and causing injuries
44. Limited key resources (e.g. feeders, drinkers, enrichment)
45. Barren environments - lack of ability to express natural behaviours (exploration, foraging, rooting, nest building at farrowing).
46. Artificial lighting regimes (e.g. lux, and length of time, etc)
47. Air quality e.g. dust
48. Air quality e.g. high conc. of atmospheric ammonia, carbon dioxide etc
49. Excessive noise levels (constant or sudden)
50. Lack of barriers/dividers in group housing pens (to avoid aggressive interactions)
51. Competitive feeding systems (e.g. floor feeding)
52. Confinement systems (e.g. farrowing crates)
53. Lack of shelter/shade for outdoor animals (thermal comfort/control)
54. No provision of wallows or dust bowls for outdoor pigs
55. Poor pasture quality for outdoor pigs
56. Lack of thermal control indoors
57. Delayed or inappropriate intervention at farrowing
58. Poor handling/stockperson skills (e.g. slapping, yelling, etc)
59. Inability of stockpeople to interpret pig behaviour (ethology)
60. Neglect – lack of regular herd inspection
61. Selection of animals suited to management system (e.g. appropriate breed or genotype)
62. Breeding for increasingly large litter sizes (ability of sow to cope?)
63. Inbreeding micro-pigs to select for smaller animals (increased risk of deformities)
64. Stress/injury caused from loading animals onto truck
65. Premature pre-transport feed withdrawal
66. Stress/injury caused by transport – long and short distances
67. Transporting vulnerable animals – specifically pregnant and cull sows
68. Domestic transport in inappropriate/substandard vehicles
69. Understocking/overstocking vehicle compartments
70. Delayed euthanasia decisions for sick/injured animals
71. Euthanasia method not carried out correctly
72. Mass depopulation (methods, and unpreparedness)
73. Tail length (too short)
74. Confinement and management of teaser boars
75. Predation (outdoor pigs)
76. Lack of vet undergraduate training on pigs
77. Piglet crushing by sows in the farrowing crate
78. Owners purchasing pet pigs without doing research of specific welfare needs
79. Too easy to buy “mini pigs” and other pet pigs online
80. Overfeeding pet pigs

*Poultry*

(n=81)

1. Lack of routine health care (e.g. vaccinations, parasite prevention, etc)
2. Poor leg & foot health (e.g. lameness, pododermatitis, hock burn, foot pad lesions)
3. Keel bone fractures & damage (laying hens)
4. Lack of medicine available specific to poultry
5. Not using antibiotics even when required (NAE,RWA)
6. Delayed veterinary care
7. Hock, breast, and foot pad burns due to contact with damp litter
8. General poor health status (e.g. common metabolic/reproductive/respiratory diseases)
9. Severe ascites (fluid in peritoneal cavity) esp. in broilers
10. Reduced feather cover (result of poor feed intake, feather pecking, etc)
11. Poor management of hypothermia
12. Neglect – lack of regular flock inspection
13. Lack of pest control /biosecurity (e.g. rodents, insect infestation)
14. Unpreparedness for emergency (e.g. fire, flood, disease outbreak, etc)
15. Practices to discourage broody behaviour
16. Delayed action to manage outbreak of feather pecking or cannibalism
17. Chronic hunger in broilers and turkey breeders
18. Inadequate provision of feed/forage
19. No continuous provision of clean water
20. Feeding a diet, or diet form, inappropriate for their ages/stage of production
21. Lack of access to insoluble grit (to aid digestion)
22. Build-up of stale/contaminated feed or water
23. Restrictive feeding regimens (e.g. to control weight gain during pre-lay)
24. Stress caused by solitary living (group species)
25. Limited key resources in housed poultry (e.g. feeders, drinkers, perches, nest boxes)
26. Group composition (unstable social groups will disrupt social hierarchy, sex ratios)
27. Insufficient space allowance (e.g. no opportunity for exercise, inability to stretch wings, etc)
28. Presence of abnormal behaviours (e.g. stereotypy, feather pecking, etc)
29. Aggression/injuries between birds
30. Cages for layers (general)
31. Lack of appropriate environmental enrichment (no ability to express natural behaviours e.g. dust bathing, litter for scratching, foraging, etc)
32. Unsuitable flooring for housed birds (e.g. causing discomfort, injury, etc)
33. Injury from on farm facilities (e.g. wire, plastic, sharp edges)
34. Lack of provision or poor quality bedding/litter
35. Artificial lighting regimes (e.g. lux, and length of time, etc)
36. Air quality - dust levels
37. Air quality - high conc. of atmospheric ammonia, carbon dioxide, etc
38. Excessive noise levels (constant or sudden)
39. Lack of continuous daytime access to open runs for outdoor (free-range) birds
40. Lack of shelter/shade for outdoor animals (thermal comfort)
41. Lack of overhead cover for outdoor birds (fear of predation)
42. Poor pasture quality (e.g. not clean/dry)
43. Predation (outdoor birds)
44. Poor handling skills e.g. carrying birds solely by the head, neck, one wing/leg, or tail feathers, injury during chick and poult processing
45. Lack of ability of stockpeople to interpret poultry behaviour (ethology)
46. Delayed removal of dead birds from cages
47. Caretakers inability to recognize poor health status / clinical signs
48. Pain caused by beak trimming (hot blade trimming)
49. Pain caused by beak treatment (infrared energy light)
50. Pain caused by retrimming adult bird beaks
51. Pain caused by dubbing broilers (removing comb or wattle)
52. Breeding for exaggerated body conformation
53. Breeding animals with inheritable defects
54. Use of 'spiking' roosters in flock aggression
55. Selection for rapid growth (link to e.g. lameness, heart disease, etc)
56. Use of appropriate breed or genotype (ability to withstand env. / climatic conditions)
57. Transport (general) – both long and short distances
58. Stress caused by gathering (pre-transport)
59. Automated handling procedures (pre-slaughter)
60. Premature pre-transport feed withdrawal
61. Fitness for transport (esp. chicks and poults)
62. Understocking and overstocking of transport boxes
63. Inappropriate handling of transport boxes (injury, dropped from heights, etc)
64. Housing facilities unprepared for receiving chicks/poults (thermal stress, hunger, etc)
65. Delayed euthanasia decisions for sick/injured birds
66. Euthanasia methods
67. Mass depopulation (e.g. methods, and unpreparedness)
68. Disposal of spent laying hens
69. Unwanted male chicks
70. Lack of vet undergraduate training on poultry
71. Mis-sexing of pet chickens (e.g. can lead to relinquishment of roosters, etc)
72. Uncleanliness of plumage
73. Purchase of birds online etc without suitable preparation/knowledge/research
74. Despurring turkeys (removing spur bud)
75. Declawing turkeys (removal of dew and pivot claw)
76. Not using turkey saddles before mating
77. Improper toe cutting of turkeys (should be done by vet > 72 hrs)
78. Desnooding turkeys (should be done by vet > 21 days)
79. Mutilation of wing tissue (inc. dewinging, pinioning, severing tendons)
80. Semen collection of tom turkeys (pain, handling, cloacal bleeding, overstimulation)
81. No provision of open water access for ducks (for drinking, bathing, and swimming)

*Cattle*

(n=72)

1. Lack of routine health care (e.g. vaccinations, parasite prevention, etc)
2. Lack of veterinary services in some areas (“cold spots”)
3. Poor health status (e.g. metabolic/reproductive/respiratory disease)
4. Common production diseases e.g. mastitis, etc
5. Common cattle infectious diseases (e.g. BVD, Johne’s, TB, etc)
6. Lack of proper foot care (e.g. regular paring, foot bathing, treatment, etc)
7. Poor foot health (e.g. white line disease, digital dermatitis, lameness, etc)
8. Underutilisation of NSAIDS
9. Exposure/ingestion of toxic substances/plants (e.g. photosensitisation)
10. High morbidity/mortality rates for calves
11. Mineral deficiency
12. Poor body condition
13. Dystocia
14. Early maternal separation (dairy)
15. Method and timing of weaning (beef)
16. Calving management & bull selection (e.g. breeding oversized calves)
17. Poor management and nutrition of orphan beef calves
18. Poor colostrum management
19. Inappropriate drying off techniques (diary)
20. Intensive finishing systems (beef) e.g. high energy finisher diets
21. Lack of fibre in diets (important for rumen health)
22. Inadequate provision of feed/forage (e.g. quantity)
23. No continuous provision of clean water
24. Build up of stale/contaminated feed or water
25. Feeding inappropriate diet to changing animal requirements (e.g. parturient cattle nutrition)
26. Limited key resources (e.g. feeder spaces, drinkers, cubicles, etc)
27. Insufficient space allowance (e.g. housed and grazed animals)
28. Group composition (e.g. unstable social groups disrupt hierarchy, sex ratios, etc)
29. Stress caused by isolation (temporary or permanent)
30. Poor maternal behaviour (cow-calf bonding)
31. Presence of abnormal behaviours (stereotypy e.g. tongue rolling, etc)
32. Aggression/injuries between cattle
33. Poor cubicle design (dairy)
34. Permanent housing (no access to pasture/grazing)
35. Poor lighting regime for housed livestock (e.g. lux, length of time, etc)
36. Poor air quality in buildings (e.g. dust, etc)
37. Lack of shelter/shade for grazing animals (thermal comfort)
38. Lack of bedding material (or poor quality) in buildings
39. Poor flooring surface and hygiene (e.g. wet, slippy, soiled)
40. Poor maintenance of equipment/building e.g. handling facilities, fencing
41. Poor pasture quality e.g. wet, muddy
42. Lack of environmental enrichment (ability to express natural behaviours) for housed animals
43. No provision of hospital/relief pen for sick animals
44. Excessive noise levels (constant or sudden)
45. Poor handling skills (e.g. tail twisting, slapping, yelling, etc)
46. Stress caused by routine handling e.g. gathering, milking, weighing
47. Lack of ability of stockpeople to interpret cattle behaviour (ethology)
48. Lack of recognition of clinical signs of poor health/disease
49. Neglect - failure to regularly inspect livestock (e.g. heifers)
50. Delayed calving intervention
51. Use and control of farm dogs (biting, stress)
52. Unskilled/incompetent service providers (e.g. foot trimmer, etc)
53. Pain caused by castration
54. Pain caused be disbudding/dehorning
55. Pain caused by electro-ejaculator
56. Pain from vasectomy
57. Pain caused by tattooing
58. Pain caused by ear notching
59. Breeding for exaggerated conformation traits e.g. double muscling
60. Breeding selection for increased production (e.g. increasingly high milk yields)
61. Use of appropriate breed/genotype for rearing environment (e.g. ability to withstand climatic conditions)
62. Reproductive management practices (use of artificial insemination)
63. Live transport (short and long distances)
64. Transport of vulnerable cattle (e.g. cull cows, pregnant cows, etc)
65. Domestic transport in inappropriate/substandard vehicles
66. Premature pre-transport feed withdrawal
67. Understocking/overstocking truck compartments
68. Delayed euthanasia decisions for sick/injured animals
69. Mass depopulation (e.g. methods, and unpreparedness, etc)
70. Unwanted male dairy calves
71. Poor pest control (bio-security) e.g. birds, rodents, insects, spreading disease
72. Presence of horns (physical injuries and blindness due to horn occlusion)

*Small Ruminants*

(n=76)

1. Lack of routine health care (e.g. vaccinations, parasite prevention, etc)
2. Poor health status (e.g. metabolic/reproductive/respiratory disease)
3. Common small ruminant health issues (e.g. myiasis, sheep scab, etc)
4. Common small ruminant infectious diseases (e.g. brucellosis etc)
5. Poor foot health (e.g. lameness, scald, foot rot, digital dermatitis)
6. Gastrointestinal parasites
7. Reduced range of suitable analgesics for small ruminants
8. Lack of veterinary services in some areas (cold spots)
9. Underutilisation of NSAIDS
10. Poor dental health (condition of molar and incisor teeth)
11. Exposure/ingestion of toxic substances/plants
12. Poor lamb/kid vigour at birth - high neonatal mortality
13. Delay in veterinary/professional engagement
14. Poor body condition
15. Dystocia
16. Poor management of orphan lambs/kids (inc. fostering and hand rearing)
17. Inadequate pest control / bio-security measures
18. Method and timing of weaning (excluding dairy goats/sheep)
19. Early separation (or temporary separation) of lambs/kids from dam
20. Tethering/restraining lactating ewes e.g. use of lamb adopter
21. Stress caused by isolation (including temporary)
22. Failing to shear mature sheep min once per year (of breeds that require it)
23. Shearing in winter (lack of protection from weather conditions)
24. Mineral deficiency
25. Poor colostrum management
26. Inadequate provision of feed/forage
27. No continuous provision of clean water
28. Feeding inappropriate diet to changing animal requirements (e.g. parturient)
29. Build-up of stale/contaminated feed or water
30. High stocking density for housed and grazed animals
31. Group composition (e.g. mixing unfamiliar sheep, sex ratios, etc)
32. Poor maternal behaviour (ewe-lamb bonding)
33. Aggression/injuries between animals
34. Permanent housing of dairy goats (no access to pasture)
35. Poor artificial lighting for housed livestock (lux, length of time, etc)
36. Poor building ventilation (air quality)
37. Lack of shelter/shade for grazing animals (thermal comfort)
38. Lack of bedding material (or poor quality) in housing
39. Poor flooring surface and hygiene (e.g. slippy, soiled)
40. Poor maintenance of equipment/buildings e.g. handling facilities, fencing, on-farm debris
41. Poor pasture quality e.g. wet, muddy
42. Lack of environmental enrichment (ability to express natural behaviours) for housed animals
43. No provision of hospital/relief pen for sick animals
44. Limited key resources for housed animals (feeding racks, drinkers, etc)
45. Excessive noise levels (constant or sudden)
46. Poor handling skills (e.g. lifting/dragging by fleece, tail, horns, etc)
47. Stress caused by routine handling e.g. gathering, milking, etc
48. Lack of ability of stockpeople to interpret sheep/goat behaviour
49. Neglect – failure to regularly check flock (e.g. cast sheep)
50. Delayed lambing intervention
51. Lack of recognition of clinical signs of poor health/disease by stockperson
52. Use and control of farm dogs (biting, stress)
53. Unskilled/incompetent service providers e.g. shearers
54. Pain caused by castration
55. Pain caused by tail docking
56. Pain caused by electro-ejaculator
57. Pain from vasectomy
58. Pain caused by ear notching
59. Pain caused by tattooing
60. Improper disbudding
61. Breeding for exaggerated body conformation (e.g. double muscling)
62. Use of appropriate breed or genotype for environment/management system (e.g. able to thrive on grass, ability to withstand climatic conditions)
63. Breeding animals with inheritable defects
64. Reproductive management practices (e.g. use of artificial insemination)
65. Transport – long and short distances
66. Transport of vulnerable animals e.g. pregnant and lame sheep/goats
67. Domestic transport in inappropriate/substandard vehicles
68. Understocking/overstocking truck compartments
69. Delayed euthanasia decisions for sick/injured animals
70. Correct fitting and maintenance of ram equipment (harness, raddle)
71. Presence of horns (e.g. physical injuries and blindness due to horn occlusion)
72. Predation
73. Unwanted male goat kids
74. Lack of vet undergraduate training on sheep/goats
75. Fleece or coat cleanliness/quality
76. Tail length (too short)
